# Diagnosis and management of a trapped lung or diaphragm by fractured ribs: analysis of patients undergoing rib fracture repair

**DOI:** 10.1186/s12893-019-0581-x

**Published:** 2019-08-28

**Authors:** Ying-Hao Su, Shun-Mao Yang, Huan-Jang Ko

**Affiliations:** 1 Department of Orthopaedics, National Taiwan University Hospital Hsin-Chu Branch, 25, Lane 442, Sec 1, Jingguo Rd, Hsinchu City, 30059 Taiwan; 2 Division of Thoracic Surgery, Department of Surgery, National Taiwan University Hospital Hsin-Chu Branch, 25, Lane 442, Sec 1, Jingguo Rd, Hsinchu city, 30059 Taiwan

**Keywords:** Trapped lung, Trapped diaphragm, Surgical stabilization of rib fracture, Rib fracture

## Abstract

**Background:**

There are few reports regarding a lung or diaphragm trapped by a fractured rib. This study aimed to describe the clinical presentations, diagnosis, and management of these intrathoracic pathologies.

**Methods:**

We retrospectively reviewed the database at our institute for patients with rib fractures who underwent thoracoscope-assisted surgical stabilization of rib fracture (SSRF). We analyzed the demographic data, mechanism of trauma, presentations, operative findings, and subsequent management strategies.

**Results:**

A total of 38 consecutive patients who underwent SSRF were analyzed. Three patients had a trapped lung and one had a trapped diaphragm. Abnormal radiographic findings were observed in 50% of cases. The median waiting time for surgery was 25 days. Surgery was indicated for intractable dynamic pain following conservative treatment. A definitive diagnosis was made during thoracoscopic exploration. Thoracoscopic repair and resection were used for trapped lungs and thoracoscopic release for a trapped diaphragm. We subsequently performed SSRF for unhealed rib fractures.

**Conclusion:**

As per our analysis, the incidence of a trapped lung or diaphragm was 10.5%. If a patient presents with persistent intractable dynamic pain, thoracoscopic exploration with concurrent SSRF may be a feasible and effective treatment option.

## Background

Blunt trauma to the chest often causes rib fractures. Severe rib fracture and flail chest can result in respiratory failure, subsequent pneumonia, and prolonged intensive care unit (ICU) stays [1, 2]. An inwardly displaced fractured rib may cause laceration to the intercostal vessels and lung resulting in hemothorax or pneumothorax. The incidence of certain rare intrathoracic pathologies, such as, lung or diaphragm laceration, and their relationship to rib fractures has been previously reported [3]. However, only two reports have discussed trapped lungs caused by rib fracture [4, 5]. There are no reports of trapped diaphragm.

The aim of the study was to describe cases with a trapped lung or diaphragm, including the mechanism of trauma, diagnosis, surgical approaches and outcomes, development of the condition over time, and presentation. We present a feasible and safe alternative approach to treat these injuries.

## Methods

The Institutional Review Board of National Taiwan University Hospital, Hsin-chu Branch—a level II trauma center—approved the study design and provided permission for this study. We retrospectively reviewed our database of patients with rib fractures who underwent thoracoscope-assisted surgical stabilization of rib fractures (SSRF). The operation notes were reviewed and patients with either a documented trapped lung or diaphragm were enrolled. Their medical records, including chief complaints, physical findings, mechanisms of trauma, and computed tomography (CT) scans of the chest were reviewed.

### Surgical techniques

**Fig. 1:**
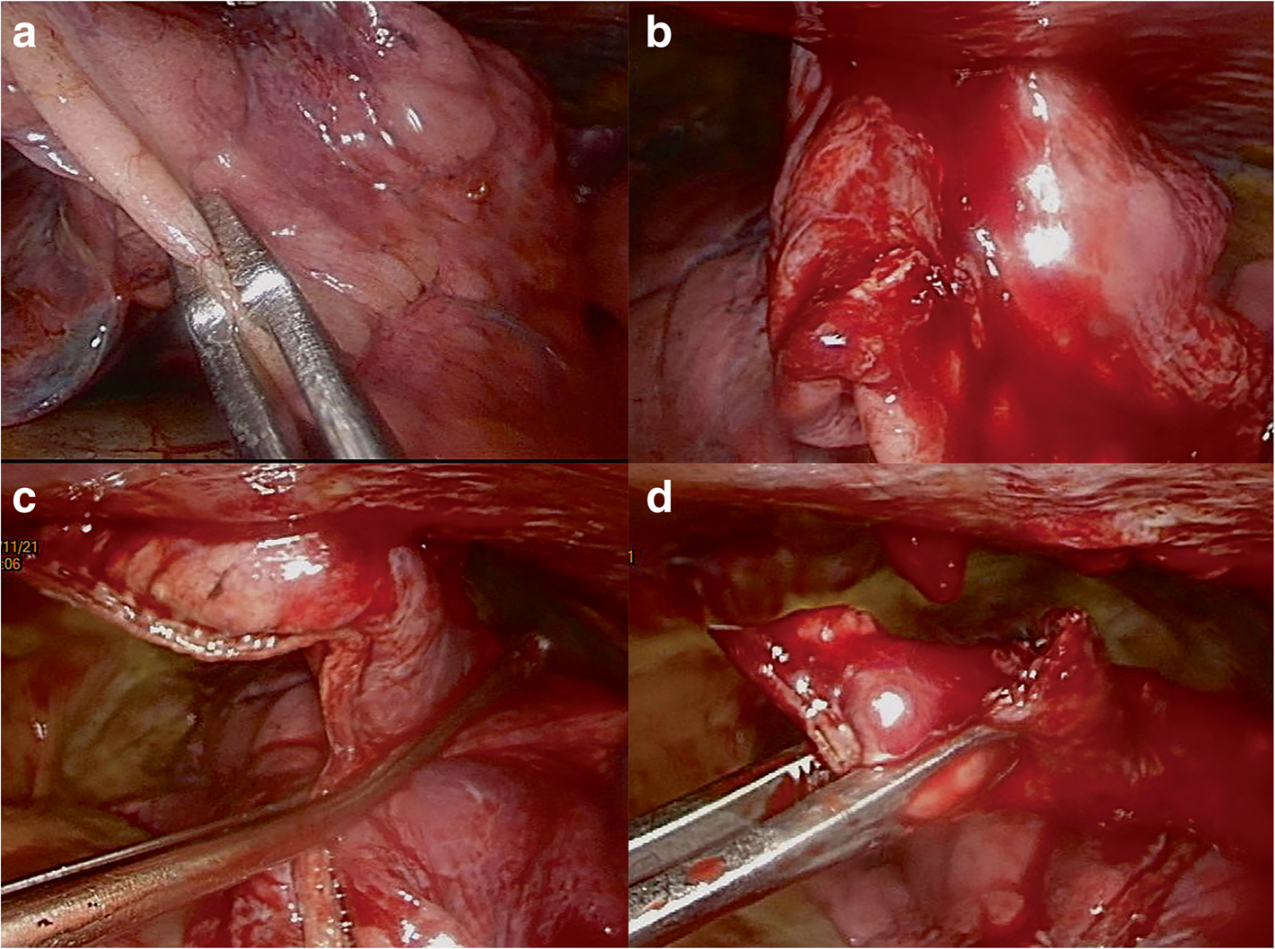
Thoracoscopic exploration shows the lung parenchyma trapped by the fractured left 6th rib (**a**). Gentle dissection was attempted, but it was obstructed by the fractured rib and increased oozing (**b**). Repair and resection was performed with endoscopic linear cutter stapler (**c**). The trapped parenchyma was released (**d**)

**Fig. 2:**
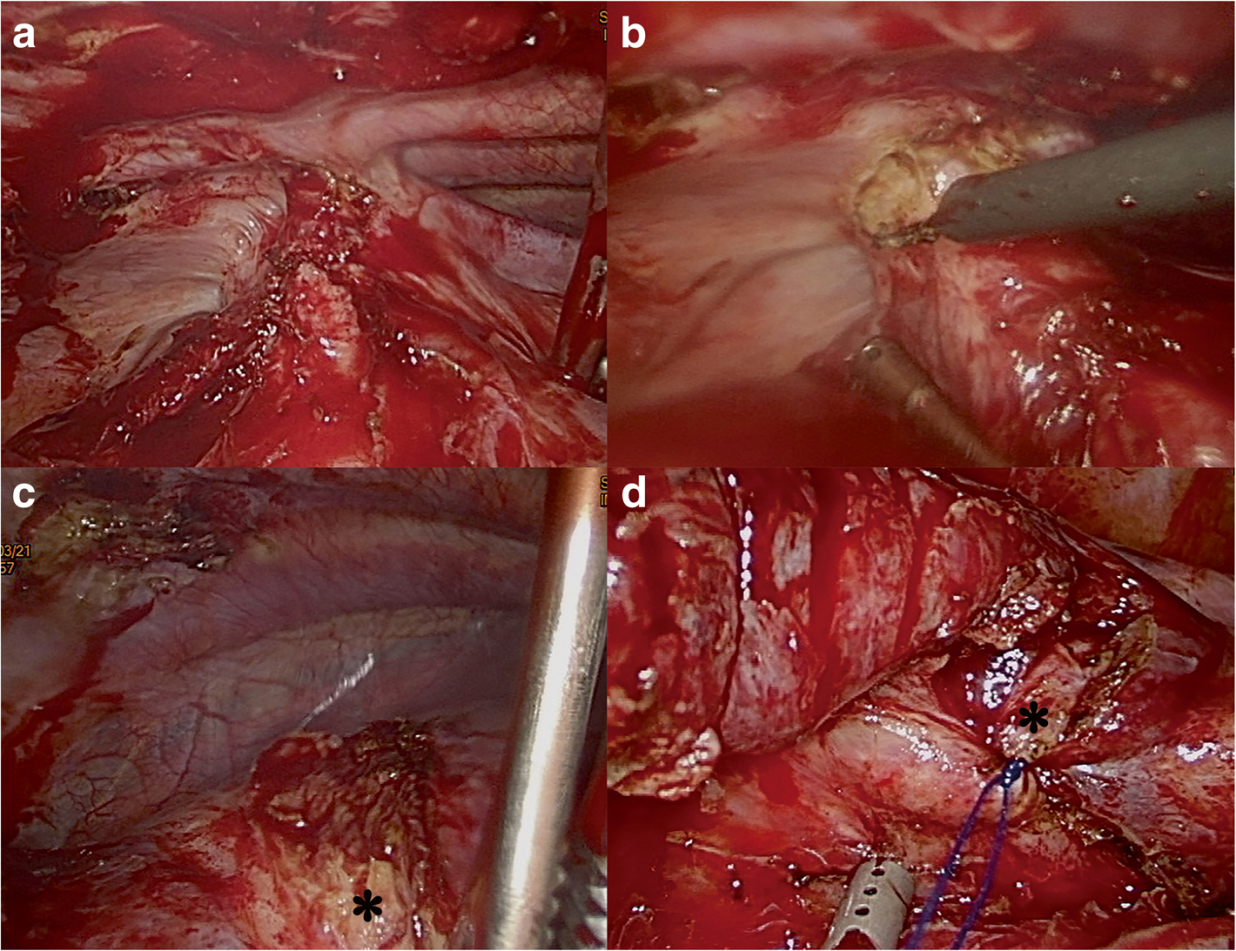
Thoracoscopic exploration shows the left diaphragm trapped by the fractured left 9th and 10th ribs (**a**). The trapped diaphragm was released by electrocautery along the inner cortices of the ribs (**b**). A defect was noted after the release of the trapped diaphragm (denoted by the asterisk) (**c**). The defect was repaired with 3–0 Prolene sutures (**d**)

Patients underwent general anesthesia with single lung ventilation using either a double-lumen endotracheal tube or a bronchial blocker prior to surgery. The patients were placed in the lateral decubitus position using flexion of the hips to increase the intercostal space. We initially performed video-assisted thoracoscopic surgery (VATS). First, the pleural space was cleaned. If the time-interval between the injury and the operation was more than 2 weeks, numerous fibrous bands developed within the pleural space. These were released by electrocautery. The resection and repair technique described by Chou et al. for the management of lung laceration was performed for a trapped lung [6]. The injured lung parenchyma was resected and repaired using an Endoscopic Linear Cutter Stapler. The resected parenchyma was removed through the VATS port **(**Fig. 1**)**. A trapped diaphragm was released along the fractured rib. Any defect in the diaphragm was noted following surgical release and was repaired using 3–0 Prolene sutures (Fig. 2). Subsequently, we used a stress test to check the stability of the fractured rib. We performed concurrent Open Reduction Internal Fixation (ORIF) with locked plates, using a muscle-sparing approach without thoracotomy for unhealed fractured ribs [7].

Patient characteristics including age, sex, mechanism of trauma, direction of force, fractures of the rib, laterality of injury, associated injuries, chest abbreviated injury scale (AIS), injury severity score (ISS) and operative data, including the time between the injury and operation, and the operated ribs were recorded. The locations of the trapped lungs or diaphragms were classified into anterior, lateral, and posterior according to the anterior and posterior axillary line [8, 9]. Clinical and radiographic results were also documented.

## Results

Between June 2016 and September 2017, 38 consecutive patients underwent SSRF at our institute. Of those, 22 sustained non-flail chest injuries and 16 sustained flail chest injuries. Three patients had trapped lungs and one had a trapped diaphragm. The incidence of a trapped lung or diaphragm was 10.5% (4/38). Data are summarized in Table 1. Only one patient with a trapped lung sustained a non-flail chest injury. The patient with a trapped diaphragm also sustained a flail chest injury. All injuries were sustained following a motor scooter collision (MSC). One patient was admitted via our emergency department (ED). Three patients were referred to our Orthopedics or Thoracic Surgery outpatient clinics for surgical consultation due to intractable dynamic chest wall pain. Two patients had associated injuries; one sustained an ipsilateral clavicle fracture and the other sustained a pelvic fracture.

**Table 1:** Patient Demographics and Surgical Results

	Patient 1	Patient 2	Patient 3	Patient 4
Lesion				
Trapped lung	Y	Y		Y
Trapped diaphragm			Y	
Demographics				
Age	48	52	62	22
Sex	M	M	M	F
Injury				
Mechanism of trauma	MSC	MSC	MSC	MSC
The direction of force	LC	LC	LC	LC
Laterality of injury	Left	Left	Left	Left
Fractured ribs	2nd-6th	4th–8th	2nd-12th	3th–5th
Trapped area	6th	6th, 7th	9th, 10th	5th
Location	Lateral	Posterior	Posterior	Anterior
Chest AIS	4	4	4	3
ISS	20	20	21	9
Surgical results				
Hospital admission	ED	OPD	OPD	OPD
Timing (day)	5	19	33	31
Operated ribs	6th	5th–7th	9th, 10th	3rd-5th
Follow-up (month)	20	18	16	10

*MSC* Motor scooter collision, *LC* Lateral compression, *AIS* Abbreviated injury scale, *ISS* Injury severity score, *ED* Emergency department, *OPD* Outpatient department

**Fig. 3:**
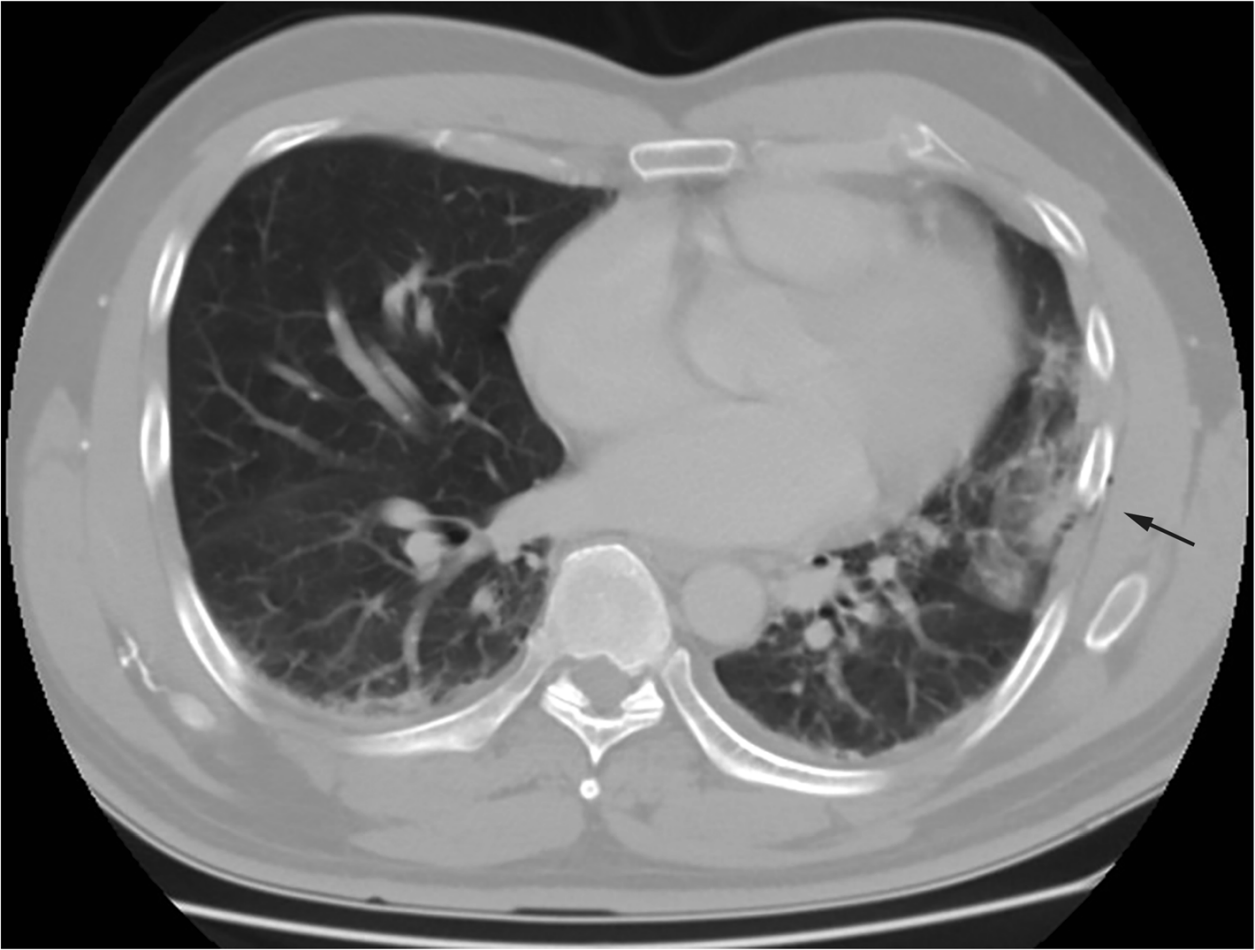
Computed tomography (CT) scan of the chest reveals lung atelectasis with intercostal extension of the lung parenchyma (arrow) secondary to being trapped by the left 6th rib

**Fig. 4:**
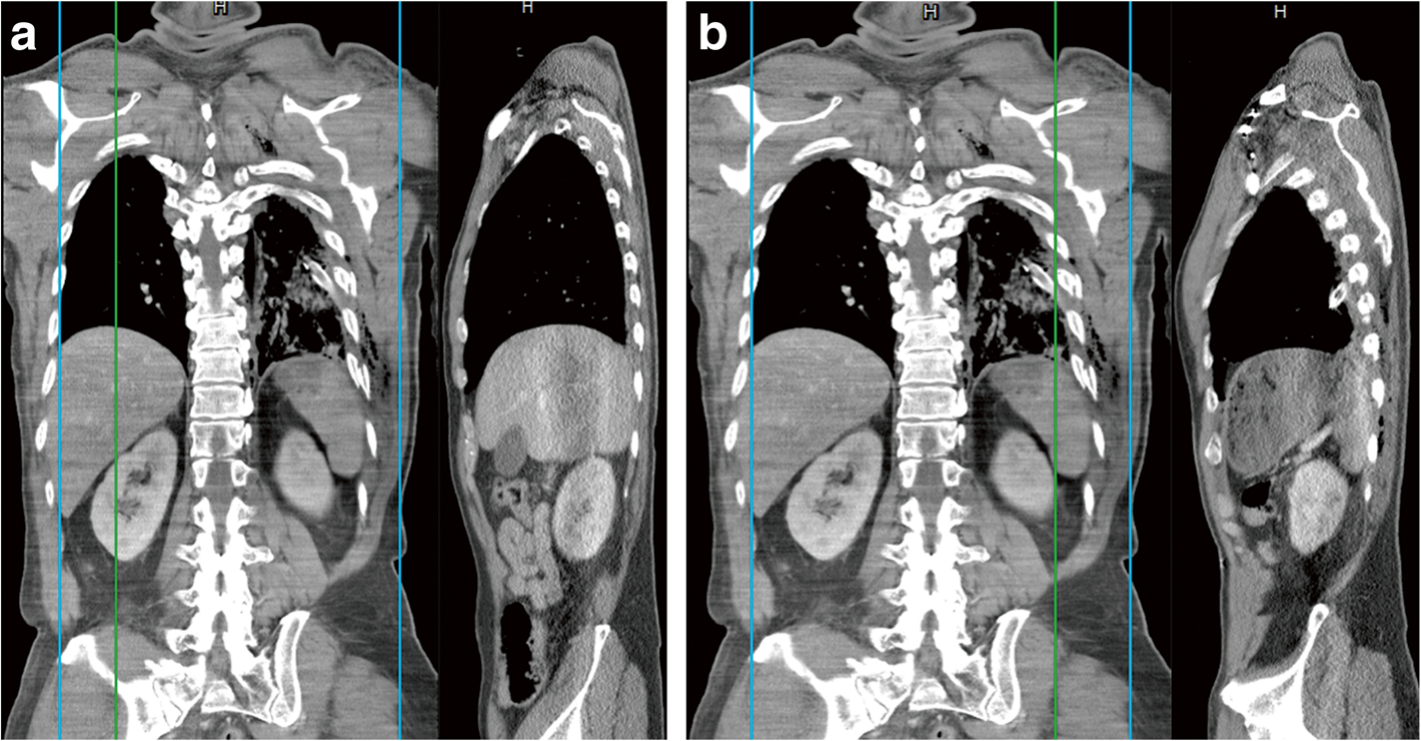
Computed tomography (CT) scan of the chest of Case no. 3 reveals normal dome shape of the right posterior diaphragm (**a**). Contralateral sagittal reconstruction of the CT scan of the chest reveals loss of the dome shape and altered contours of the left posterior diaphragm secondary to being trapped by the fractured left 9th and 10th ribs (**b**)

Patients experienced minimal pain at rest and no respiratory problems. However, all patients experienced severe and intolerable dynamic pain. Chest pain was out of proportion to their rib fractures following 5–33 days of conservative treatment. Patients had to remain in recumbent postures, e.g., in a wheelchair or lying on their back. The pain in their chest walls was related to the movement of the shoulder and was not resolved with the use of analgesics. On physical examination, they complained of fixed and aggravated chest wall pain caused by passive movement of the ipsilateral shoulder. Chest CT revealed lung atelectasis with intercostal extension of the lung parenchyma in only one patient (Fig. 3). The chest CT scan of the other 2 patients with trapped lungs only showed displaced ribs with unhealed fractures. Sagittal reconstruction of the chest CT scan in the patient with a trapped diaphragm showed loss of the normal dome shape of the left posterior diaphragm (Fig. 4).

The median duration between injury and surgery was 25 days. The patients with trapped lungs underwent repair and resection. The patient with a trapped diaphragm underwent thoracoscopic release. Following release of the trapped lung or diaphragm, a stress test was used to assess the stability of the fractured ribs. Rib fractures were unhealed following VATS. All four patients underwent concurrent SSRF. Postoperative recovery was uneventful, with no major perioperative complications such as delayed hemothorax/pneumothorax, implant failure or breakage, or wound infection. One patient complained of a frequent dry cough after surgery, which was relieved using oral mucolytics. All patients reported significant improvements in dynamic pain. The median duration of follow-up was 17 months. Administration of oral analgesics ceased one month after surgery. Follow-up radiography showed healed ribs without loosening, breakage, or migration of the implants. All patients were satisfied with the outcome of their surgery.

## Discussion

Fracture of the ribs is common in patients with blunt chest trauma, which can result in pneumothorax, hemothorax, and laceration of the lung. Some rare intrathoracic pathologies, e.g., parenchyma laceration, diaphragm/esophagus laceration, were reported by Holcomb et al. [3]. However, a review of literature revealed that reports of traumatic trapped lung or diaphragm following blunt chest trauma are rare. To the best of our knowledge, only two reports presented two cases of trapped lung following the fracture of a rib. Okubo et al. reported this rare condition, including the mechanism of trauma, radiographic findings, management, and comments from an expert [4]. Schots et al. reported one case of captured lung in a series of 15 patients who underwent SSRF [5]. One was diagnosed by CT scan of the chest and the other case was diagnosed by VATS performed prior to ORIF.

There were 3 cases of trapped lung and 1 case of a trapped diaphragm in our series. The incidence was 10.5% (4/38). One patient presented to our ED in an acute stage and underwent VATS and SSRF on the fifth day of injury. The other 3 patients were referred to our Orthopedics or Thoracic Surgery outpatient clinics for surgical consultation and underwent VATS and SSRF 19–33 days after sustaining the injury. None of the patients experienced respiratory problems. Chest radiographs revealed no pneumothorax or hemothorax. Patients experienced intolerable dynamic pain of the chest wall. Dynamic pain was aggravated by motion of the ipsilateral shoulder. Lung atelectasis with intercostal extension of the parenchyma or loss of the double-domed contour of the diaphragm is a common radiographic abnormality that was observed in 50% of cases. A definitive diagnosis can made by VATS exploration.

All patients suffered a lateral compression injury following an MSC. Our hypothesis is that a trapped lung or parenchyma can result from a lateral impact that is not too severe, such as an MSC. The thoracic cage is a semi-rigid ring structure and the sternocostal, costotransverse and costovertebral joints are relatively mobile. When a rib is laterally compressed, it can be fractured and twist inwards causing elongation of the intercostal muscles. However, the energy is not severe enough to disrupt and strip both the intercostal muscle and parietal pleura; therefore, when the force ceases, the fractured rib may pinch the lung or diaphragm and return outwards partially due to the pull of the intercostal muscle, resulting in a trapped lung or diaphragm. Repeated inflammation and tethering during motions of the chest wall or ipsilateral shoulder may contribute to dynamic pain. Dynamic pain may also be caused by incarceration of an intercostal nerve or the parietal pleura by fractured ribs and/or a trapped lung or diaphragm. Fabricant et al. reported that entrapment of the intercostal nerve was found in 38% of 24 patients with painful rib fracture nonunion [10] and they performed neurolysis for suspected intercostal nerve entrapment. The parietal pleura contains sensory fibers responsive to painful, tactile, and thermal stimuli. The incidence of these lesions may be underestimated since a trapped lung may compress the lung laceration and the patient may not have hemothorax or pneumothorax. The patients in this study did not present with respiratory failure. Without respiratory problems, most surgeons are taught that the pain and disability of rib fractures resolves in 6–8 weeks [11]. However, if a trapped lung or lesion is left untreated, nonunion may develop as the fracture gap is interposed with soft tissues. Patients may therefore suffer from prolonged pain and disability.

We prefer repair and resection for management of a trapped lung. Okubo et al. described the successful management of a trapped lung using thoracoscopic release without SSRF in the acute stage [4]. Schots et al. reported using thoracoscopic release with SSRF [5]. Most of our patients were not in the acute stage; they underwent VATS between 5 and 33 days following injury. A longer wait between injury and treatment may lead to severe fibrosis and adhesions between the lung and the fractured rib. Forced dissection with a sharp instrument or a cotton dissector may cause iatrogenic lacerations of the parenchyma in the subacute stage. Furthermore, a trapped lung may have a laceration of lung parenchyma, potentially resulting in bleeding, air leakage, or longer intubation. Therefore, repair and resection may be a safe and effective alternative.

SSRF is effective in the management of rib fractures. Previous reports have established that it has several advantages in flail chest injuries including reduced rates of mechanical ventilation and tracheostomy and reduced duration hospitalization [12–14]. Patients with intractable pain following non-flail chest injuries are candidates for SSRF [15–17]. In our series, most patients complained of chronic intractable dynamic pain with disturbances to activities of daily life and they were unable to return to their jobs. Following the treatment of the interposed trapped lung or diaphragm by thoracoscopic release, the stress test showed no solid union of the fractured ribs. We preferred concurrent SSRF as the treatment of choice for such unhealed rib fractures. In our study, the fractures were not in the acute stage and the fracture site was interposed with the parietal pleura, lung, or diaphragm. Fibrosis occurred in cases where there was no contact between the segments of the fracture. Adequate debridement of the interposed fibrous tissue and stable fixation with locked plates is more reliable as it provides better pain relief and results in higher rates of union in unhealed fractures of the rib.

This study has certain limitations. This was a retrospective single-center study with a small number of patient and no control group. All the patients sustained lateral compression injuries following MSCs; therefore, the mechanism of trauma may be different from that of other reports. There is still no reliable tool for preoperative diagnosis of trapped lung or diaphragm. Indications for surgery and ideal timing for patients with intractable dynamic pain following rib fractures is still lacking.

## Conclusion

The incidence of a trapped lung or diaphragm was 10.5% (4/38) in our series. Although patients did not present with respiratory failure, they experienced intractable dynamic pain with disturbed function of the ipsilateral shoulder. Abnormal radiographic findings were observed in 50% of the patients. A surgical diagnosis was mandatory for such intrathoracic pathologies. Repair and resection for a trapped lung and surgical release for a trapped diaphragm are both feasible and safe. Concurrent ORIF for rib fractures can stabilize the chest wall; it may also help to achieve bone union and facilitate the process of recovery.

## Abbreviations

AIS: Abbreviated injury scale
CT: Computed tomography
ED: Emergency department
ICU: Intensive care unit
ISS: Injury severity score
MSC: Motor scooter collision
ORIF: Open reduction internal fixation
SSRF: Surgical stabilization of rib fractures
VATS: Video-assisted thoracoscopic surgery
